# A New Approach of Complexing Polymers Used for the Removal of Cu^2+^ Ions

**DOI:** 10.3390/polym16070920

**Published:** 2024-03-27

**Authors:** Nicoleta Mirela Marin

**Affiliations:** 1 National Research and Development Institute for Industrial Ecology ECOIND, Street Podu Dambovitei No. 57-73, District 6, 060652 Bucharest, Romania; nicoleta.marin@incdecoind.ro; 2 Department of Oxide Materials Science and Engineering, National University of Science and Technology Politehnica Bucharest, 1–7 Gh. Polizu, 060042 Bucharest, Romania

**Keywords:** complexing agent, maize stalk, Amberlite IRA 402 (Cl^−^), complexing mechanism, copper ions, removal

## Abstract

This study presents two modified polymers for Cu^2+^ ion removal from aqueous media. Shredded maize stalk (MC) and a strong-base anionic resin (SAX) were modified with indigo carmine (IC) in order to obtain two different complexing polymers, i.e., IC-MC and SAX-IC. Initially, the complex reaction between IC and Cu^2+^ in the solution was studied. Additionally, the complex formation Cu^2+^-IC in liquid solutions was evaluated at different pH ranges of 1.5, 4.0, 6.0, 8.0, and 10.0, respectively. For Cu^2+^ ions, adsorption onto the IC-MC and IC-SAX batch experiments were conducted. The contact time for evaluating the optimum adsorption for Cu^2+^ ions on the complexing materials was established at 1 h. Efficient Cu^2+^ ion adsorption on the IC-MC and SAX-IC at pH = 10 was achieved. The adsorption of Cu^2+^ ions depends on the quantity of IC retained on MC and SAX. At 2.63 mg IC/g MC(S4) and 22 mg IC/g SAX(SR2), a high amount of Cu^2+^ ion adsorption was reported. The highest adsorption capacity (*Q_e_*) of IC-MC was obtained at 0.73 mg/g, and for IC-SAX, it was attained at 10.8 mg/g. Reusability experiments were performed using the HCl (0.5 M) solution. High regeneration and reusability studies of IC-MC and IC-SAX were confirmed, suggesting that they can be used many times to remove Cu^2+^ ions from aqueous matrices. Therefore, the development of complexing materials could be suitable for Cu^2+^ ion removal from wastewater.

## 1. Introduction

Water pollution is currently one of the most hotly debated problems worldwide [1]. As a solution, green technologies represent promising alternatives in wastewater depollution [2]. The challenges and future perspectives integrate the promising role of green technologies in the removal of existing inorganic contaminants from wastewater [3]. For this purpose, green technologies provide promising alternatives in wastewater depollution processes [4,5,6]. At the same time, current trends use vegetable materials as well as conventional resins for retention following a modification process in order to improve their adsorption capacity [7]. It is known that the hazardous impacts of heavy metals on aqueous media persist over time, affecting aquatic processes and leading to drastic effects on human health due to their toxicity and non-degradability mechanisms [8]. Adsorption based on complexing interactions is an efficient method in terms of design simplicity and ease of operation to solve the problems of hazardous metal removal [9]. Consequently, many papers have been published on the development of chelating adsorbents over the years [10]. In general, the preparation of chelating adsorbents includes two aspects: the matrix, which can be a material obtained by synthesis or a natural matrix, and modification agents [11,12]. Since the functional groups of chelating agents largely dominate the selectivity and adsorption capacity of new chelating materials developed, the surface of modified adsorbent becomes one of the important research aspects [13,14]. Also, complexing agents possess functional groups containing donor atoms that are capable of complexing metal ions. Thus, complexing resins have been used in environmental applications due to their selectivity and specificity for metal ion removal [15,16,17]. At the same time, an essential characteristic of complexing materials is their stability in different environments [18,19,20]. In this regard, synthetic samples were prepared in order to reproduce the environmental conditions and were tested on complexing materials [21,22]. In the literature, studies are presented that show the adsorption of analytical reagents on cellulosic material as well as on ion-exchange resin [23,24]. Modified polymers using complexing agents have the following advantages regarding metal ion removal: (i) chemical modification can be carried out using synthetic and natural polymers rapidly compared to conventional complexing polymers obtained by synthesis methods, where traditional complexing resins require special complexing agents, but most of these agents are hard to commercially employ, especially since they are expensive and sometimes difficult to synthesize [25,26]; (ii) the efficiency of complexing polymers for metal ion removal can be improved by optimizing the experimental conditions; (iii) the regeneration of these polymers is possible after metal ion adsorption. Also, after the adsorption process, metal ions can be recovered from the surface of complexing polymers; and (iv) complexing polymers can be reused, making the adsorption process more economical. However, complexing polymers also have disadvantages, as most laboratory research may face impediments related to the fact that private investors are only interested in innovations that can reach TRLs 5–6 (technology readiness levels) in order to obtain quick results for recovering the investment made [27].

In the present study, a variant was chosen in which a polymeric support (natural/synthetic) and an organic reagent with complexing properties are required to obtain complexing polymers. For this purpose, an anion exchange resin and natural polymer represented by maize stalk were used as polymer supports due to their structure capable of retaining organic reagent with complexing proprieties via physical and chemical interactions.

**Figure 1:**
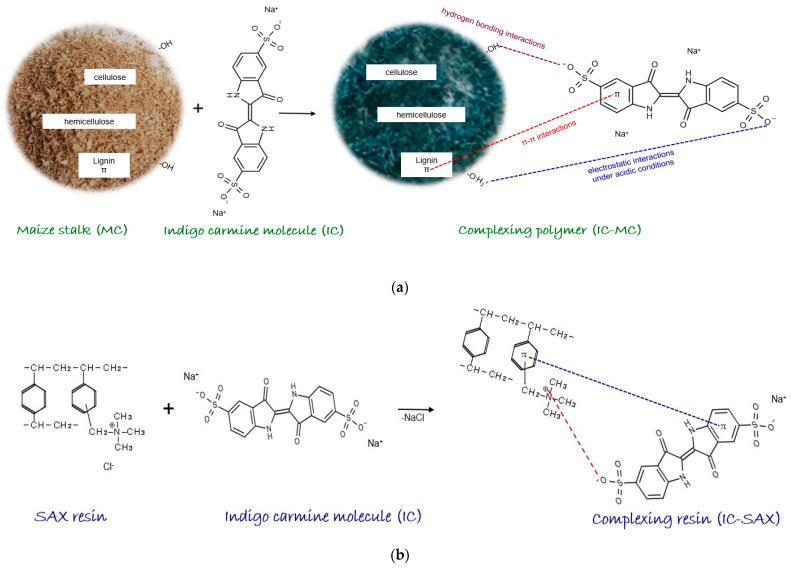
Adsorption of IC and MC (**a**) and SAX resin (**b**).

MC is a complex natural polymer composed of lignin (~14%), cellulose (37–39%), and hemicellulose (30–35%) as major components, along with lipids, sugars, and proteins in variable proportion, containing a variety of functional groups such as hydroxyl, carboxyl, amino, sulfhydryl, etc. [28,29]. The proposed interaction between IC and MC is illustrated in Figure 1a. For IC adsorption on MC, the following interactions can be proposed [27,30,31,32,33,34,35]. Hydrogen bonding interactions may be involved under the following conditions: MC contains –OH groups while IC has O (strong H donor). The proposed hydrogen bonds occur between the strong H donor (O) of IC and the H acceptor of MC. Additionally, electrostatic interactions may be involved when the negatively charged sulfate molecules interact with positively charged –OH_2_^+^ groups from the MC structure, which can be obtained under acidic conditions when –OH groups are protonated with H^+^, resulting in –OH_2_^+^. Consequently, electrostatic interactions are probably involved when protonated hydroxyl groups of MC and SO_3_^−^ groups of IC in acidic condition reactions. π-π interactions take place between the π electrons from the hemicellulose structure and the π electrons of IC from the benzene structure. All these interactions contribute to IC adsorption on the natural polymer maize stalk [29,36]. Also, IC is soluble in aqueous solutions, and sulfonic groups from its structure are ionized, converting IC into anionic organic compounds. The subsequent adsorption process is obtained via the interaction between anionic IC compounds and the functional groups of the SAX resin Cl^−^ form (R-N^+^(CH_3_)_3_Cl^−^). Therefore, it is expected that the IC will be adsorbed on SAX polymeric resin by an ion exchange equilibrium, as depicted in Figure 1b, along with π-π interactions that are manifested between the aromatic rings of styrene divinylbenzene and the aromatic groups of the complexing agent. Considering that there are no literature data available regarding the use of MC or SAX resin to obtain complexing materials using IC as a complexing agent, a new experimental study was carried out. This research aimed to contribute novelty and originality to the field of biotechnology for wastewater treatment polluted with toxic Cu^2+^ ions by developing a fast and eco-friendly technology based on complexing polymers. To achieve this goal, the following experimental parameters were studied: (i) the influence of contact time to establish an optimal time for the adsorption of Cu^2+^ ions on the completing materials, (ii) the influence of pH solution to obtain optimum adsorption of Cu^2+^ ions on the IC-MC and SAX-IC, (iii) the influence of IC quantity retained on MC and SAX for Cu^2+^ ions removal, (iv) the influence of Cu^2+^ ions concentration on complexing materials, and (v) the regeneration and reuse of exhausted complexing polymers with Cu^2+^ ions for testing their sustainability.

## 2. Materials and Methods

### 2.1. Materials

All chemicals used here are of analytical grade. A styrene-divinylbenzene matrix with quaternary ammonium functional group in Cl^−^ form, specifically Amberlite IRA 402 (Cl^−^), was employed to obtain complexing resin with IC (Sigma Aldrich, St Louis, MO, USA). The complexing agent, indigo-5,5′-disulfonic acid disodium salt, 37% HCl, and certified reference materials (MRC) consisting of 1000 mg/L Cu(NO_3_)_2_, Pb(NO_3_)_2_, and Ni(NO_3_)_2_ were purchased from Merck (Lowe, NJ, USA). Maize stalk was collected at maturity and processed for application in batch experimental studies. The same procedures as presented in previous studies [7] regarding collecting, shredding, purification, and activation were applied to the obtained shredded maize stalk with a particle size of 1 mm.

### 2.2. Apparatus

A UV-Vis spectrophotometer, model DR/5000 TM (Hach Lange, Berlin, Germany), was employed to determine the concentration of the complexing agent at a fixed wavelength (λ) selected at λ = 615 nm. A mechanical stirrer, model GFL 3017, was used to stir the mixed samples for adsorption studies. The pH of the buffer solutions and aqueous matrices was checked using the pH meter, model HI 255 Combined Meter (pH/mV and EC/TDS/NaCl) from Hanna Instruments (Ann Arbor, MI, USA). Atomic Absorption Spectroscopy (AAS) was performed using a Perkin Elmer (Waltham, MA, USA) apparatus operating in air/acetylene flame mode for the analysis of metal ions. The mass of the samples was measured using an XT220A analytical balance from Precisa Gravimetrics, Dietikon, Switzerland, with a precision of ±0.0001 g.

### 2.3. Experimental Methodology

In this paper, all experimental studies were conducted in duplicate, and only the average value was utilized to present the experimental data from Section 3. Additionally, synthetic samples were prepared to replicate the environmental conditions and to test the complexing polymers obtained.

### 2.4. Linearity of Spectrophotometric Method

**Figure 2:**
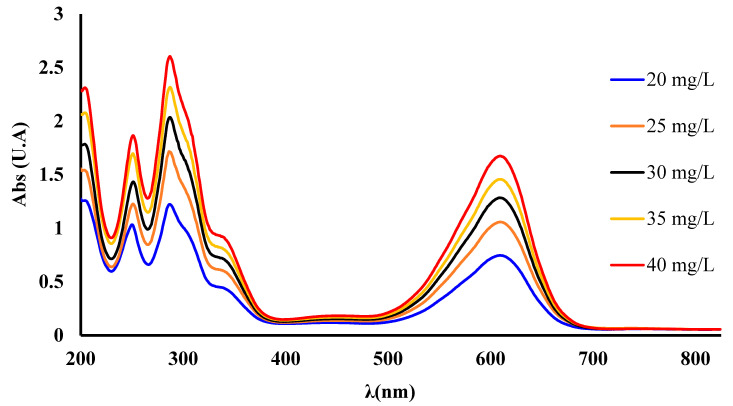
UV-Vis spectra of IC recorded in the 200–800 nm range for a study linearity of the spectrometric method.

The linearity of the spectrometric method was studied to assess the concentration of the complexing agent employed in modified polymers and is presented in Figure 2. For this purpose, the calibration curve was evaluated at five points with concentration ranges of 20, 25, 30, 35, and 40 mg/L IC, respectively. From the graphical representation of the absorbance (A) vs. concentration (C), the equation of straight line was determined to be A = 0.0400C − 0.1534, and the correlation coefficient (R^2^) had a value of R^2^ = 0.9995. The high value of R^2^ suggests good linearity of the UV-Vis method over the entire concentrations range studied, indicating that Beer’s law was checked.

### 2.5. Linearity of the AAS Method

The linearity of the AAS method was verified over a concentration range varying from 1 to 5 mg. Solutions for establishing the linearity of the AAS method were prepared from a 1000 mg/L MRC solution of Cu(NO_3_)_2_. Initially, a working solution was prepared as follows: 5 mL of the MRC solution (1000 mg/L) was quantitatively transferred into a 50 mL volumetric flask (to create the working solution). Subsequently, the solutions for drawing the calibration curve were obtained by diluting the working solution to obtain the following concentrations of 1. 0, 2.0, 3.0, 4.0, and 5 mg/L, respectively. The wavelength utilized to detect Cu^2+^ ions was 232.75 nm. In order to draw the calibration line, each standard solution was analyzed. The calibration line, representing absorbance intensity versus concentration in mg/L, was determined as follows: A = 0.1099C + 0.0159 with R^2^ = 0.9994.

Also, the determination limit (LD) for assessing Cu^2+^ ions was quantified at 3.5 µg/L, applying the same methodology described in previous studies [7].

### 2.6. Procedures for Complex Evaluation

The optimal conditions for complex evaluation between Cu^2+^ and IC at different pH solutions were identified. To achieve this, 1 mL of IC (0.3 mg/L) was mixed with 1 mL of a buffer solution with pH values of 1.5, 4.0, 6.0, 8.0, and 10.0, respectively, along with 0.5 mL of Cu^2+^ (50 mg/L). The mixture was then placed in a spectrometer cuvette, and UV-Vis spectra were recorded for each solution obtained after 10 min of reaction in the 200–825 nm range. Note: the solution with pH = 1.5 was obtained by adding drops of 1 M HCl to ultrapure water. Also, 0.1 M citrate buffer pH = 4.0, 0.1 M acetate buffer pH = 6, 0.1 M phosphate buffer pH = 8, and 0.1 M carbonate buffer pH = 10 were employed in the spectrometric studies.

### 2.7. Methodologies Used for Obtained Complexing Materials

#### 2.7.1. Methodology for Obtaining the MC-IC

To determine the adsorption capacity of MC with the complexing agent, the following experimental conditions were applied. Initially, 0.2 g of MC was stirred for 1 h at 175 rpm (T = 25 ± 2 °C) with 0.01 L IC solution (pH = 9.30) at different concentrations varying from 20, 30, 40, and 50 to 60 mg/L. At the end of the stirring time, the samples were filtered, and the solutions were collected in Erlenmeyer flasks. The quantity of complexing agent adsorbed on MC mass (*m*) was evaluated by applying Equation (1).

(1) $$Q_{e}=\frac{\left(C_{i}-C_{e}\right)V\;}{m}$$

where *C_i_* is the initial concentration of IC (mg/L), *C_e_* is the concentration of IC that remained in solution at equilibrium (mg/L), *Q_e_* is the quantity of IC adsorbed per unit of the MC mass, *V* is the solution volume (L), and m is the quantity of MC (g).

#### 2.7.2. Methodology for Obtaining the IC-SAX

In this study, SAX resin was conditioned in an acidic solution, applying the same methodology described in the previous study [21]. For obtaining the complexing resin, 0.2 g samples of dry SAX resin were placed in Erlenmeyer flasks, to which 0.01 L IC solutions (pH = 9.30) were added with the following concentrations: 32, 96, 176, 300, and 400 mg/L. The mixtures were stirred for 1 h at 175 rpm (T = 25 ± 2 °C). At the end of the stirring time, the modified resin was filtered, and solutions were collected to analyze the quantity of IC remaining in the supernatant solutions.

### 2.8. Evaluation of the Polymers Loaded with IC Stability

The stability of chelating materials was evaluated in acidic and basic solutions. A total of 0.1 g from each complexing polymer was stirred with 0.02 L of 0.5, 1.0, 1.5, and 2.0 M HCl and NaOH, respectively, for 3 h at 175 rpm (T = 25 ± 2 °C). Each sample was filtered, and the supernatant solution was analyzed spectrometrically to evaluate the quantity of IC released from the mass of modified polymers. Also, desorption studies of IC released from the mass of IC-MC and IC-SAX were conducted to test their stability, and this was calculated using Equation (2).

(2) $$D\;(\%)=\frac{A_{1}}{A_{2}}\times 100$$

where *A*_1_ is the IC quantity (mg) released in the filtrate solution after desorption studies, and *A*_2_ is the IC quantity (mg) that remains in the materials’ mass after the desorption (*D*) experiment.

### 2.9. Procedure Applied for Evaluating the Kinetics of Cu^2+^ Adsorption on the Complexing Materials

Samples of 0.05 g IC-MC and IC-SAX were weighed on an analytical balance and transferred into Erlenmeyer flasks. Then, 0.01 L of carbonate buffer solutions (0.1 M) containing 2.5 mg/L Cu^2+^ ions were added to the dry IC-MC samples, while 50 mg/L Cu^2+^ ions existing in carbonate buffer solutions were placed over the IC-SAX samples. The mixtures were stirred for varying times ranging from 10, 20, 30, 40, 50, and 60 min at 175 rpm (T = 25 ± 2 °C). At the end of the stirring time, samples were filtrated. Subsequently, from all filtrate samples, the concentration of Cu^2+^ ions that were not retained on the complexing polymers was determined by AAS. The quantity of Cu^2+^ ions adsorbed at time (*t*) on the mass of complexing material *Q_t_* (mg/g) was determined using Equation (3).

(3) $$Q_{t}=\frac{\left(C_{i}-C_{t}\right)V\;}{m}$$

where *C_i_* is the initial concentration of Cu^2+^ ions (mg/L), *C_t_* is the concentration of Cu^2+^ ions (mg/L) at time (*t*), *Q_t_* is the quantity of Cu^2+^ ions adsorbed per unit of the mass of the complexing polymer at time (*t*), *V* is the solution volume (L), and *m* is the mass of complexing polymers (g).

### 2.10. Methodologies Used for Studies Quantity of Complexing Agent Loaded on MC and SAX for Determined Optimum Cu^2+^ Ions Adsorption

Samples weighing 0.05 ± 0.0001 g contained the following amounts of IC per gram of MC: S1 (1.28 mg IC/g MC), S2 (1.73 mg IC/g MC), S3 (2.19 mg IC/g MC), and S4 (2.63 mg IC/g MC). Similarly, for SAX, the amounts of IC per gram of SR were as follows: SR1 (12 mg IC/g SAX), SR2 (22 mg IC/g SAX), SR3 (37 mg IC/g SAX), and SR4 (44 mg IC/g SAX). Additionally, non-modified polymers MC (S5) and SAX (SR5) were stirred at 175 rpm (T = 25 ± 2 °C) for 1 h with 0.01 L volumes containing 5 mg/L Cu^2+^ ions at pH 10. After 1 h of stirring, the mixtures were filtered, and the concentration of Cu^2+^ ions was determined by AAS.

### 2.11. Methodology Used to Evaluate the Influence of pH on Cu^2+^ Ions Adsorption onto Modified Polymers

Samples of 0.05 g IC-SAX (22 mg IC/g SAX) and respective IC-MC (2.63 mg IC/g MC) were weighed and transferred into Erlenmeyer flasks. Subsequently, 0.01 L buffer solution (pH = 1.5, 4.0, 6.0, 8.0, and 10) containing 3 mg/L Cu^2+^ was added to the IC-MC, while 5 mg/L Cu^2+^ was added to the IC-SAX samples. The buffer solutions used were citrate buffer solution for pH = 4.0, acetate buffer solution for pH = 6.0, phosphate-buffered solution for pH = 8.0, and carbonate buffer for pH = 10.0, respectively. To obtain pH = 1.5, the mixture solution containing Cu^2+^ ions and IC was treated with a few drops of HCl 0.1 M.

### 2.12. Procedure for Adsorption of Cu^2+^ on IC-MC and IC-SAX Materials

For this experiment, samples weighing 0.05 g of IC-MC (2.63 mg IC/g MC) and IC-SAX (22 mg IC/g SAX) were stirred using 0.01 L solution pH = 10, with different Cu^2+^ concentrations at 175 rpm and at T = 25 ± 2 °C. For Cu^2+^ adsorption on IC-MC, the following concentrations were used: 2.5, 3.0, 3.5, 4.0, 4.5, 5.0, 5.5, 6.0, 6.5, and 7.0, respectively. Also, for Cu^2+^ adsorption on IC-SAX, the concentration range used was 5.0, 6.0, 7.0, and 50.0 to 58.0 mg/L. The removal efficiency (*R* %) of Cu^2+^ ions on the solid mass was determined using Equation (4):

(4) $$R\;(\%)=\frac{\left(C_{i}-C_{e}\right)}{C_{i}}\times 100$$

where *C_i_* denotes the initial concentration of Cu^2+^ ions (mg/L), and *C_e_* denotes the concentration of Cu^2+^ that exists in the solution at equilibrium (mg/L).

### 2.13. Procedure for Regeneration of Complexing Materials after Cu^2+^ Ions Adsorption and Reuse Experiments

For the regeneration experiment, 0.01 L of 0.5, 1.0, 1.5, and 2.0 M HCl solutions were added to 0.05 g of 0.70 mg Cu^2+^/g IC-MC and 1.3 mg Cu^2+^/g IC-SAX with Cu^2+^ ions. Subsequently, the mixtures obtained were stirred for 30 min at 175 rpm (T = 25 ± 2 °C). Each mixture was then filtered, and the liquid phases obtained were analyzed using the AAS technique utilizing the previously presented calibration curve for Cu^2+^ ion determination, as presented in Section 2.5. Also, the solid phases resulting from the regeneration experiment were kept and employed in a new adsorption–desorption experiment. For a new adsorption cycle, 0.01 L (7 mg/L Cu^2+^) was added to evaluate the recycled phases of IC-MC and IC-SAX obtained after the release of Cu^2+^ ions.

### 2.14. Procedures for Application of MC-IC and SAX-IC in Competitively Adsorption

An amount of 0.05 g of MC-IC and SAX-IC was put in each Erlenmeyer flask, with 0.01 L of tap water and tannery wastewater added. The experiment was carried out at 175 rpm (T = 25 ± 2 °C) for 1 h. Similarly, for tannery wastewater application, the same experimental procedures were applied. To reproduce wastewater samples, tap water samples were spiked with Cu^2+^, Ni^2+^, and Pb^2+^ ions in the following procedure: 5 mL of tap water was mixed with 5 mL of carbonate buffer at pH = 10, and metal ions were spiked to obtain an initial concentration detected as being 4.6 ± 1.2 mg/L Ni^2+^, 4.9 ± 1.5 mg/L Pb^2+^, and 5.2 ± 0.55 mg/L Cu^2+^. Samples of tannery wastewater (5 mL tannery wastewater + 5 mL carbonate buffer pH = 10) were analyzed, and initial concentrations were found to be 1.24 ± 0.88 mg/L Ni^2+^; 0.79 ± 1.7 mg/L Pb^2+^, and 1.72 ± 0.95 mg/L Cu^2+^. The results presented represent the mean of two duplicate experiments for metal analysis, together with their standard deviation.

## 3. Results and Discussion

### 3.1. Experimental Conditions for Complex Evaluation

**Figure 3:**
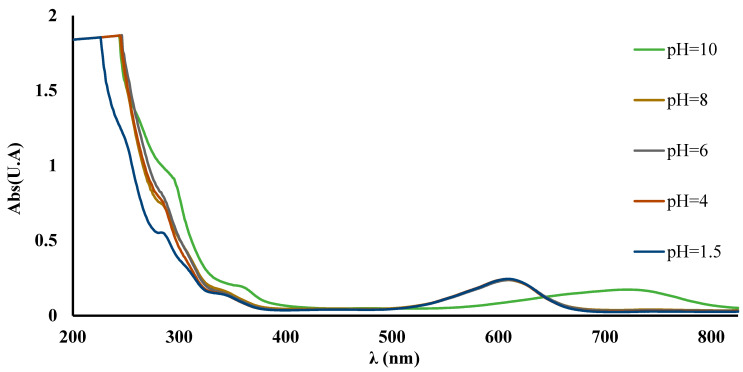
UV-Vis spectra recorded for Cu^2+^-IC obtained at different pH values.

Starting from the hypothesis that there may be a similarity between the complex behavior of Cu^2+^ ions with IC in solution and the behavior of the modified polymers (IC-MC and SAX-IC), the complex reaction in the mixture between Cu^2+^ and IC was studied. The absorption spectra of Cu^2+^ ions with the complexing agent IC at pH 1.5, 4.0, 6.0, 8.0, and 10 are shown in Figure 3.

As shown, the spectra recorded for the solutions containing Cu^2+^ ions and IC in buffer solution varying from pH 1.5 to 8.0 did not exhibit any spectral changes. However, a spectral change is only registered at pH = 10, where Cu^2+^ is complexed in the presence of IC. It was found that the pH of the aqueous solution influences the formation of the complex, as shown in Figure 3. Structural changes occur following the interaction of the metal ion with the chelating agent, as evidenced by the disappearance of the maximum at 625 nm and the appearance of a new maximum at 730 nm. Also, the literature data show that the IC forms a color complex with Cu^2+^ ions in 0.1 M carbonate buffer at pH 10.0 [37].

**Figure 4:**
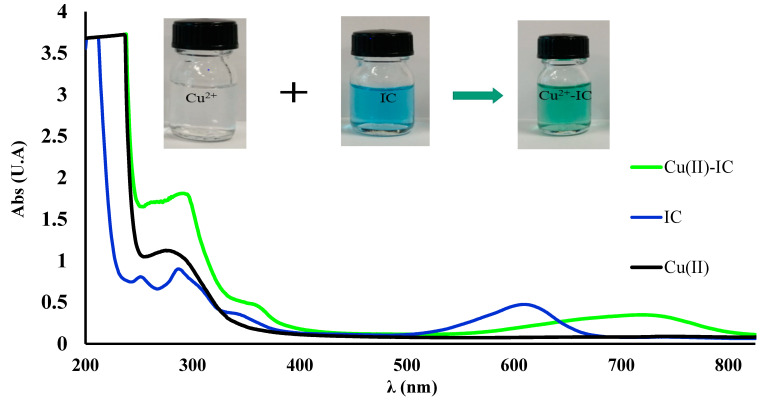
UV-Vis spectra recorded for Cu^2+^, IC, and mixture between Cu^2+^ and IC at pH = 10.

Also, another indication of complex formation for spectra is given in Figure 4, where the color of the solution obtained turns green. At the same time, it was observed that the solution of the IC-Cu^2+^ complex had maximum absorbance after 10 min of reaction at 715 nm and did not suffer any change in the spectrum during 3 h of monitoring at laboratory temperature. However, taking into account all these observations, the subsequent studies regarding the applications of the modified polymers will be based on the aspects, of the adsorption of Cu^2+^ on IC-MC, and SAX-IC will always be evaluated after 10 min of reaction.

**Figure 5:**
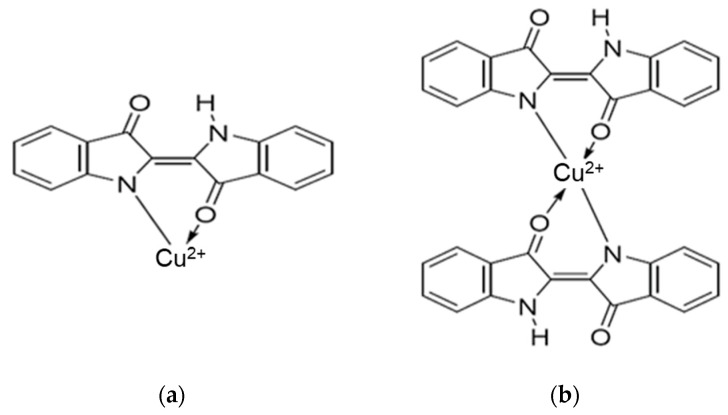
Possible mechanism for complex formation of Cu^2+^-IC with a complex ratio of 1:1 (**a**) and 1:2 (**b**).

Also, a possible complex mechanism between Cu^2+^ ions and the IC ratio is presented in Figure 5, where (i) if a single metal ion interacts with one molecule of IC, a combination ratio of 1:1 is obtained, resulting in complexes with the structure presented in Figure 5a and (ii) if one Cu^2+^ ion interacts with two molecules of ligand, an equilibrium may exist in which both molecules of IC may simultaneously combine with Cu^2+^ ion, resulting in complexes with a 1:2 combination ratio, as shown in Figure 5b.

### 3.2. Adsorption of IC onto MC and SAX Polymers

Adsorption is a measure of the interaction between solute and adsorbent by measuring the adsorption capacity (*Q_e_*) of the tested adsorbent.

**Figure 6:**
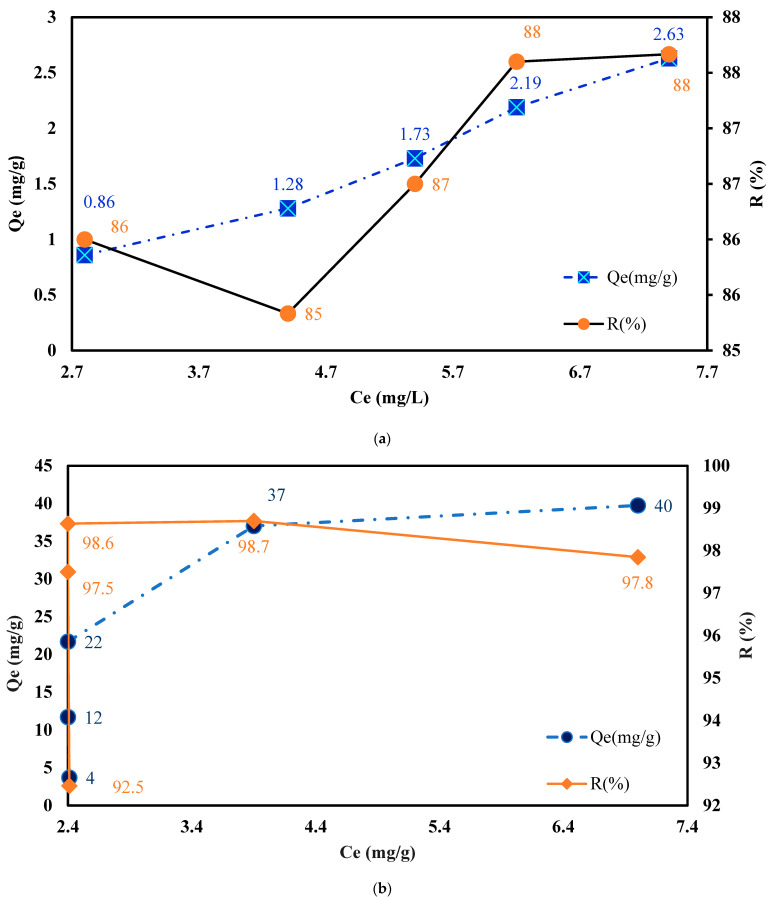
Influence of initial concentration for complexing agent adsorption onto MC *C_i_* = 20, 30, 40, 50, and 60 mg/L IC (pH = 9.3), 0.2 g MC, 1 h, and 175 rpm (T = 25 ± 2 °C) (**a**) and SAX *C_i_* = 32, 96, 176, 300, and 400 mg/L IC, 0.2 g SAX, 1 h, and 175 rpm (T = 25 ± 2 °C) (**b**).

In this paper, the first step involved modifying MC and SAX materials with IC and applying the batch method. The adsorption process was achieved by gradually increasing the initial concentration of IC, ranging from 20 to 60 mg/L IC, for MC. It was found that good adsorption of the MC depended on the *C_i_* (mg/L) of IC under the following experimental conditions: pH = 9.3 (values obtained when IC salts are dissolved in ultrapure water), 0.2 g MC, 0.01 L with varying *C_i_* (20, 30, 40, and 50 to 60 mg IC/L), and stirring at 175 rpm (T = 25 ± 2 °C) for 60 min. The quantity of IC retained on the MC gradually changed with the increase in *C_i_* (see Figure 6a). Therefore, the most significant adsorption of IC (*Q_e_* = 2.63 mg/g) was obtained at the highest *C_i_* of 60 mg/L of IC (Figure 6a).

For the second polymeric material employed, the complexing agent manifests a huge affinity, as presented in Figure 6b. In this case, the adsorption mechanism for IC on SAX resin is governed by an ion exchange mechanism that occurs between the two sulfonic acid groups of the complexing agents and the functional groups –N^+^(CH_3_)_3_ of the polymeric resin.

The adsorption of IC on SAX resin increases as *C_i_* (mg/g) increases from 32, 96, 176, and 300 to 325 mg/L. The maximum adsorption capacity of SAX for IC adsorption was found to be 40 mg/g.

In order to employ the complexing materials in the following steps, it is necessary to test their stability in acidic and basic mediums, as the majority of wastewater has acidic or basic pH.

After the adsorption studies, the stability of the chelating materials was tested. This was carried out spectrometrically by measuring the concentration of the complexing agent in the supernatant solution when 2 M HCl and Na(OH) were added. Good stability of IC-MC and IC-SAX was observed in the supernatant solutions, with IC being detected below the quantification limits of the spectrometric method. Also, the LOQ was found to be 0.35 mg/L, a value determined by applying the same methodologies presented in a previous study [21].

Therefore, the complexing materials obtained by absorbing the complexing agent onto MC or SAX have several advantages, including high complex adsorption capacity, low reagent consumption, short processing time, and excellent stability in acidic and basic matrices. These factors reduce manufacturing costs.

### 3.3. Influence of Contact Time on the Adsorption of Cu^2+^ Ions on the MC-IC

It is well known that the adsorption process is influenced by many experimental parameters, with kinetics being one of them. Kinetics processes indicate the effect of observable parameters on the global speed of the adsorption process [34].

In order to evaluate the optimal time required for effective Cu^2+^ ions adsorption from aqueous matrices onto solid phases, various contact times were tested. These intervals varied from 10, 20, 30, 40, and 50 to 60 min for both complexing polymers employed.

**Figure 7:**
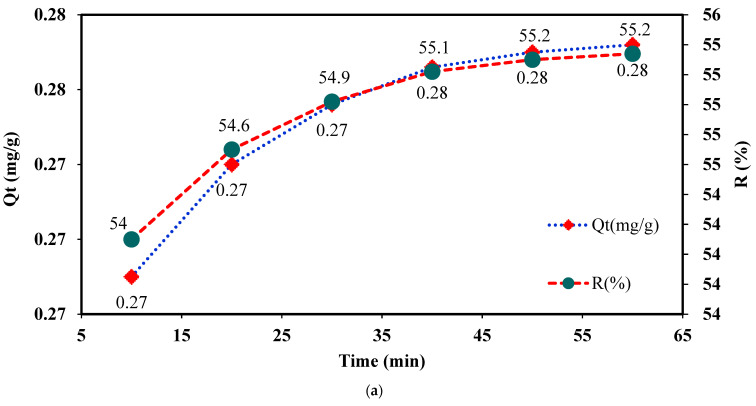

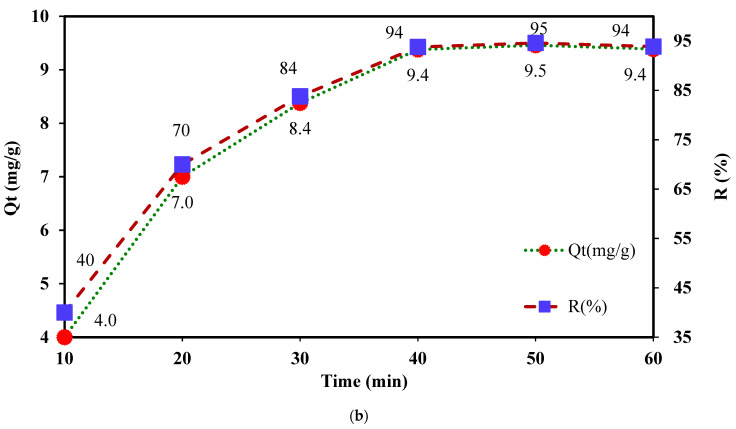
Influence of contact time for Cu^2+^ removal onto IC-MC (**a**) and IC-SAX (**b**). Experimental conditions used: 0.05 g (IC-MC and IC-SAX), contact time = 10, 20, 30, 40, 50, and 60 min, 175 rpm (T = 25 ± 2 °C), 0.01 L carbonate buffer (0.1 M) pH = 10, *C_i_* = 2.5 mg/L Cu^2+^, m = 2.63 mg IC/g MC, and *C_i_* = 50 mg/L (pH = 10) Cu^2+^ 0.01 L, m = 22 IC/g SAX.

The effect of interaction time on Cu^2+^ adsorption onto IC-MC is presented in Figure 7a. From the data obtained, it can be seen that within the first 10 min of contact between IC-MC and Cu^2+^ ions, the adsorption exceeded 50% of the *C_i_* = 2.5 mg/L Cu^2+^ initially present in the solution, which was retained in the mass of the modified material. Also, the *Q_t_* (mg/g) values determined between 20 and 60 min varied insignificantly from 0.27 to 0.28 mg/g. These data allow us to say that Cu^2+^ adsorption is fast, with significant adsorption efficiency observed within the first 20 min of contact.

For IC-SAX, it was observed that the adsorption of Cu^2+^ ions increased from 4 mg/g within the first 10 min to 9.40 mg/g at 40 min, remaining constant up to 60 min (Figure 7b). To conclude, the significant parameter of contact time that influences batch adsorption experiments was studied. In order to set a balance between liquid and solid phases, an efficient contact time for Cu^2+^ ions removal was evaluated every 10 min for 1 h while keeping the other operational parameters (mass, *C_i_*, pH medium, temperature, and scanning speed) constant. It was observed that the equilibrium state is reached quickly for MC-IC, whereas for IC-SAX resin, it was necessary to double the time for this approach.

Taking into account the porous structure of IC-MC and the increased difficulty of diffusion inside the pores, we extended the contact time to 1 h for both IC-MC and IC-SAX.

### 3.4. Influence of Complexing Agent Loaded on MC and SAX for Cu^2+^ Adsorption

It is known that the quantity of the complexing agent present in materials’ mass improves the adsorption capacity for metal ion adsorption [38]. Taking this consideration into account, the quantity of complexing agent loaded on MC and SAX was studied for the adsorption of Cu^2+^ ions from aqueous media.

For this aims, samples (S1–S4) of IC-MC with 1.28 mg IC/g MC (S1), 1.73 mg IC/g MC (S2), 2.19 mg IC/g MC (S3), and 2.63 mg IC/g MC (S4) and IC-SAX samples with 12 mg IC/g SAX (SR1), 22 mg IC/g SAX (SR2), 37 mg IC/g SAX (SR3), and 44 mg IC/g SAX (SR4), and also each material non-modified MC (S5) and SAX (SR5) were tested for Cu^2+^ ions adsorption. Increasing the IC quantity on the polymeric materials resulted in the following influence on Cu^2+^ ions adsorption.

The adsorption capacity of samples S1–S5 followed the order 0.70 mg/g (S4) > 0.68 mg/g (S3) > 0.66 mg/g (S2) > 0.64 mg/g (S1) > 0.35 mg/g (S5).

**Figure 8:**
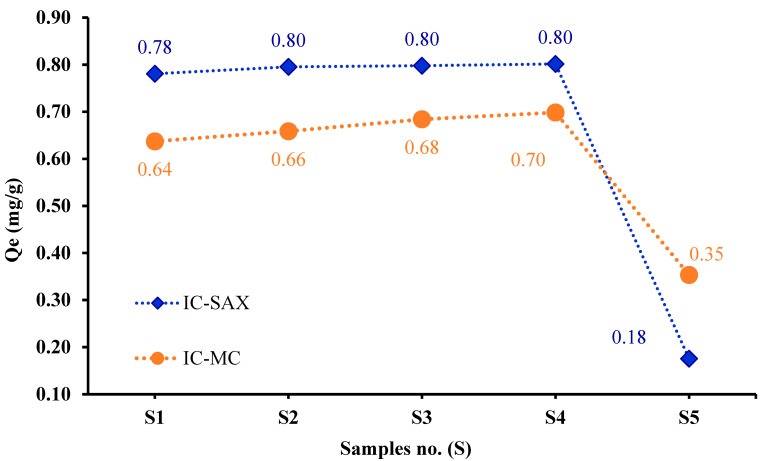
Influence of IC ratio for Cu^2+^ ions adsorption onto IC-MC and IC-SAX. Experimental conditions used: *V* = 0.01 L, *C_i_* = 5 mg/L, pH = 10, 1 h, 175 rpm T = 25 ± 2 °C, m = 0.05 g.

These results suggest that a high quantity of IC retained on the MC resulted in better removal of Cu^2+^ ions, as shown in Figure 8.

The adsorption of Cu^2+^ ions on IC-SAX was studied by varying the quantity of IC loaded on SAX masses, as previously presented for samples SR1–SR5. The quantity of Cu^2+^ ions increased from 0.78 mg/g (SR1) to 0.80 mg/g for SR2, after which the quantity of Cu^2+^ ions adsorption remained constant for samples SR3–SR4 (Figure 8). This behavior is more evident for IC-MC compared to IC-SAX

Therefore, it was observed that the complexing agent significantly influences the adsorption capacity for both developed materials compared to unmodified polymers MC (S5) and SAX (SR5) with IC.

To conclude, the adsorption capacity of MC and SAX loaded with IC increased Cu^2+^ ions adsorption as a result of the increase in functional groups that exist in the IC structure. As is presented in Figure 8, the quantity of complexing agent fixed on MC and SAX masses has an influence on metal ions adsorption. Lower values of *Q_e_* were obtained for unmodified MC and SAX compared to modified polymers for Cu^2+^ ions adsorption, suggesting adsorption via complexing interactions.

To examine the following experimental parameters, samples with 2.63 mg IC/g MC (S4) and 22 mg IC/g SAX (SR2) were used.

### 3.5. Cu^2+^ Adsorption in Function of pH Medium on Complexing Materials

**Figure 9:**
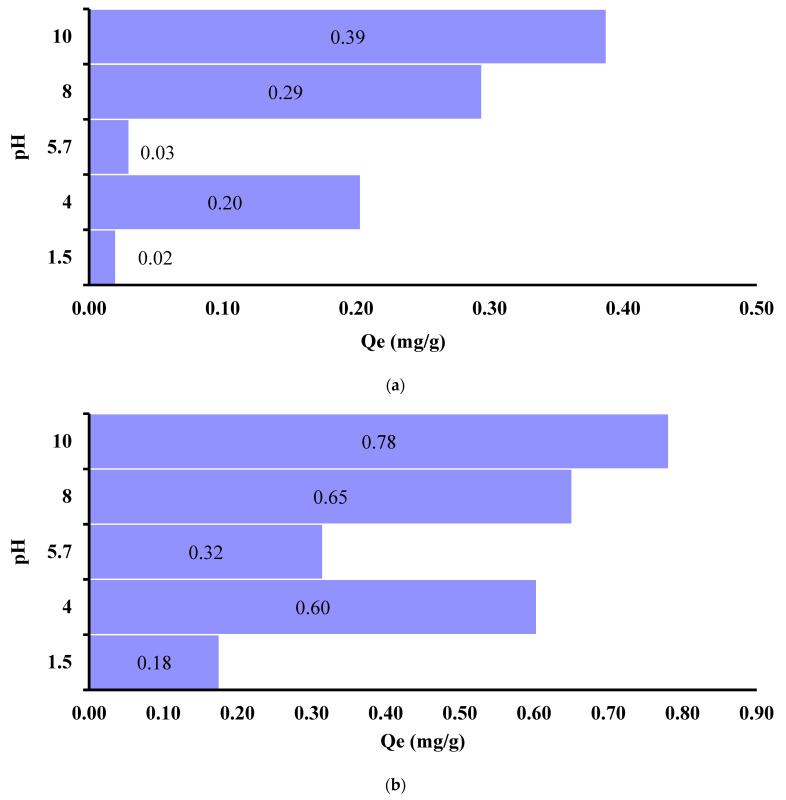
Effect of pH medium on Cu^2+^ adsorption onto IC-MC (**a**) and IC-SAX (**b**). Experimental conditions used: m = 0.05 g, *C_i_* = 3 mg/L Cu^2+^ for MC and *C_i_* = 5 mg/L for SAX, pH = 10, *V* = 0.01 L, 1 h, 175 rpm (T = 25 ± 2 °C).

Using batch techniques, the degree of Cu^2+^ ions adsorption on IC-MC and IC-SAX at pH = 1.5, 4.0, 5.7, 8.0, and 10 was determined. The aim was to evaluate if the complex between ion IC loaded on MC and SAX and Cu^2+^ ions is influenced by the pH of the aqueous matrix. By analyzing the results presented in Figure 9a,b, it can be seen that at pH = 1.5 for IC-SAX resin and at pH = 5.7 for IC-MC, low adsorption of Cu^2+^ ions was determined. Moreover, the maximum value was recorded at pH = 10 for both materials IC-MC and IC-SAX. So, this behavior is in concordance with the complex formation of Cu^2+^-IC in solution when at the same pH value where the complex was obtained. Those results suggest that the adsorption mechanism is mainly conducted via the complex mechanism between Cu^2+^ ions via the carbonyl and amino groups.

To conclude, the highest adsorption capacity of modified polymers (IC-MC and SAX-IC) for Cu^2+^ ion removal is influenced by the pH of the solution. So, to obtain a maximum adsorption capacity for Cu^2+^ ion removal onto IC-MC and IC-SAX, a medium with pH = 10 will be necessary.

### 3.6. Cu^2+^ Ions Adsorption onto IC-MC and IC-SAX

From the multitude of analytical reagents used in the spectrometric determination for metal ion complex, IC was chosen for complexing polymeric materials, taking into consideration the following reasons: (i) IC has an aromatic structure that can be retained on the polymeric resin via physical bonds; (ii) at the same time, it contains two sulfonic groups that can be retained via an ion exchange equilibrium by the anion exchange resin. At the same time, IC contains amino functional groups that contain a non-participating pair of electrons as well as carboxyl groups that can contribute to the complex formation with metal ions in well-established certain experimental conditions of pH. At the same time, the functional groups that contribute to complex formation must have an affinity for metal ions employed in the experiment; (iii) another criterion involves metal ion recovery from complexing polymers without losing their complexing properties; (iv) at the same time, IC was chosen because, in certain pH conditions, it can be selective for Cu^2+^ ion complexing [37].

**Figure 10:**
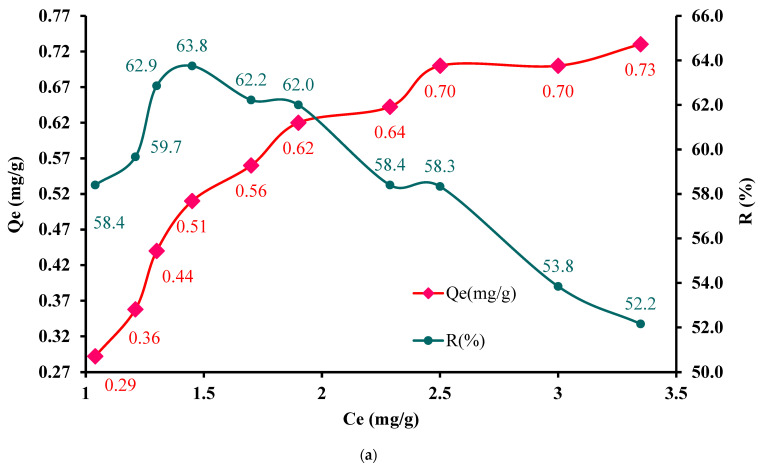

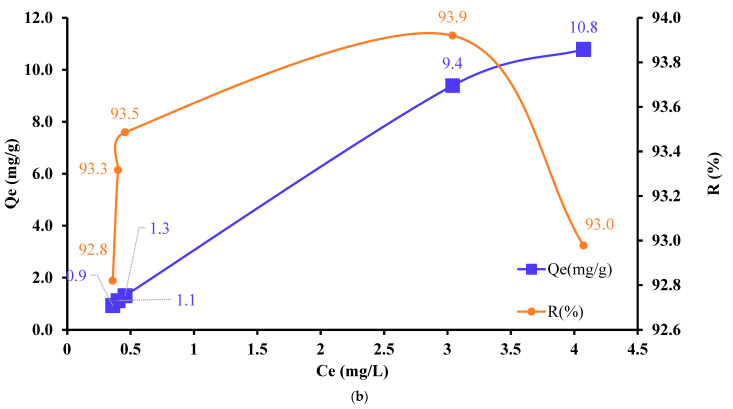
Experimental isotherm for Cu^2+^ adsorption on IC-MC (**a**) and IC-SAX (**b**). Experimental conditions used: 0.05 g (2.63 mg IC/g MC and 22 mg IC/g SAX), *V* = 0.01 L, pH = 10, 175 rpm (T = 25 ± 2 °C), *C_i_* = 2.5; 3.0; 3.5; 4.0; 4.5; 5.0; 5.5; 6.0; 6.5, 7.0 for IC-MC and *C_i_* = 5.0; 6.0; 7.0; 50.0, 58.0 mg/L for IC-SAX.

In order to test the applicability of the IC-MC material for Cu^2+^ ion removal, aqueous matrices were enriched with the following values: *C_i_* = 2.5, 3.0, 3.5, 4.0, 4.5, 5.0, 5.5, 6.0, 6.5, and 7 mg Cu^2+^/L. Significant adsorption was obtained at high metal concentrations when *Q_e_* was 0.73 mg/g (Figure 10a).

Also, to evaluate the equilibrium state between IC-SAX and Cu^2+^ ions, experimental studies were carried out in the concentration range of 5.0, 6.0, 7.0, 50, and 58 mg/L Cu^2+^ on IC-SAX functionalized resin in an alkaline pH medium. From the data presented in Figure 10b, it can be seen that with the increase in Cu^2+^ concentration, the quantity of metal ions retained on the modified resin masses increases. At the same time, this increase is more pronounced for the first *C_i_* = 5 mg/L studied, starting to reach equilibrium at the last two concentration values of 50 and 58 mg/L Cu^2+^.

Therefore, increasing *C_i_* (mg/L) of the Cu^2+^ ions in the evaluated concentration range was determined to demonstrate an increase in IC-MC adsorption capacity from 0.29 to 0.73 mg/g and for the IC-SAX resin from 0.9 to 10.8. On the other hand, the retained percentages R (%) had the following values, which decreased from 63.8 to 52.5% for IC-MC and from 93.9 to 92.8% for SAX-IC, suggesting a quantitative retention of the Cu^2+^ in both developed materials (Figure 10a,b). The affinity of Cu^2+^ adsorption on complexing polymers could be attributed to the complexing effect. The amino and carbonyl groups can complex very well with metallic ions, especially Cu^2+^ ions. So, the adsorption capacity of IC-MC and IC-SAX showed an increasing tendency as the Cu^2+^ ions concentrations increased.

In Table 1, very little research data on the removal of Cu^2+^ ions using chemical modification of natural and synthetic polymers are presented.

### 3.7. Regeneration and Reutilization Studies

It is known that when complexing material exceeds its retention capacity, it is considered that the material is exhausted and must be regenerated for a new adsorption/desorption cycle [46]. Also, once the complexing materials are exhausted, Cu^2+^ ions from the solution tested are not retained, and this aspect is monitored by the measurement in supernatant solutions of metal ion concentrations. The exhausted material may be regenerated with HCl solutions in different proportions in order to elute the metal ions adsorbed on the mass of complexing material subjected to regeneration and recover it for a new adsorption/desorption cycle.

**Figure 11:**
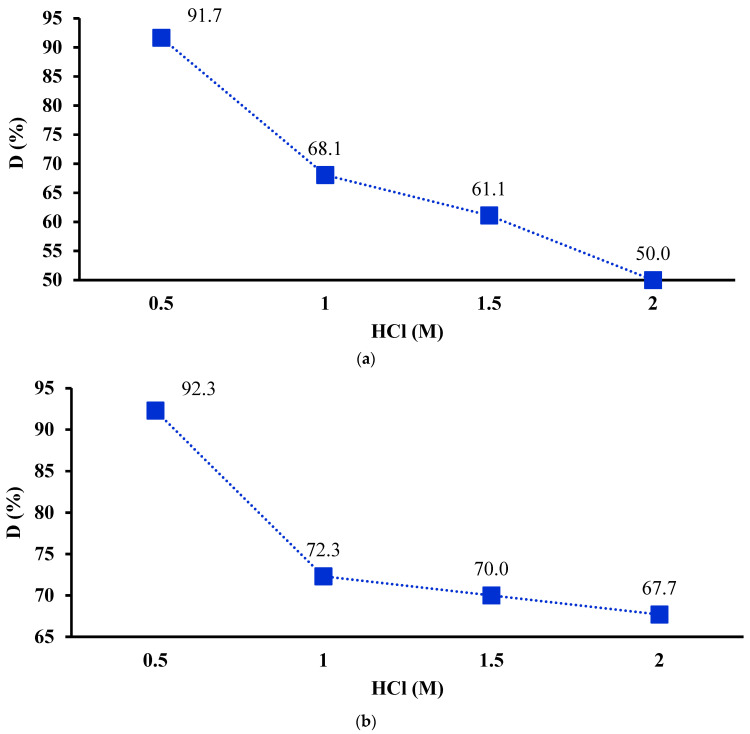
Percentages of Cu^2+^ ions desorbed from IC-MC (**a**) and from IC-SAX (**b**). Experimental conditions used: 0.05 g of 0.70 mg Cu^2+^/g IC-MC and 1.3 mg Cu^2+^/g IC-SAX, 0.01 L HCl (0.5, 1, 1.5 and 2 M), contact time 30 min, 175 rpm (T = 25 ± 2 °C).

In this regard, the chelated materials exhausted with Cu^2+^ ions were subjected to a desorption experiment using HCl solutions of 0.5, 1.0, 1.5, and 2 M. For desorption studies, solid samples loaded with 0.70 mg Cu^2+^/g IC-MC and 1.3 mg Cu^2+^/g IC-SAX were employed. The obtained results are presented in Figure 11a,b. The desorption of exhausted IC-MC with Cu^2+^ ions was found to be 91.7% when a 0.5 M HCl solution was used for regenerated solid phases loaded with 0.70 mg Cu^2+^/g MC-IC. But at the same time, a decrease in percentage release from 68.1% to 61.1% to 50% was obtained when 1.0, 1.5, and 2 M HCl solutions were used.

For IC-SAX resin loaded with metal ions, samples with 1.3 mg Cu^2+^/g IC-SAX were taken into consideration. Herein, the efficient desorption of Cu^2+^ ions was 92.3% using 0.5 M HCl > 72.3% (1.0 M HCl) > 70.0% (1.5 M HCl) > 67.7% (2.0 M HCl). Also, if we refer to the desorption percentage (%) of Cu^2+^ ions for both tested materials, a better desorption rate was obtained for SAX-IC than for IC-MC.

Taking into consideration the results obtained, a decrease in desorption efficiency was found at 2.0, 1.5, and 1 M HCl solutions than at 0.5 M HCl solution.

**Figure 12:**
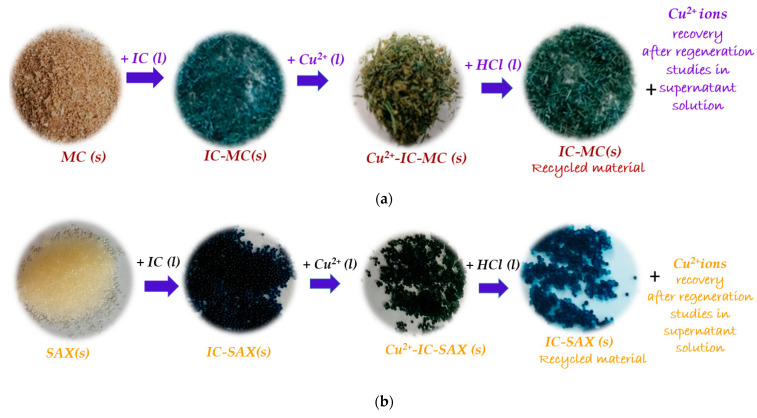
Images of MC (**a**) and SAX (**b**) materials before and after IC adsorption and subsequent complexing materials were employed in Cu^2+^ ions adsorption/desorption studies.

Also, from the images of solid materials presented in Figure 12a, it can be seen that IC-MC, after the Cu^2+^ ions release, returns to its initial color, an aspect that is highlighted for chelating material visually and by detecting Cu^2+^ ions in the regeneration solutions. The same behavior was observed for the second material tested (Figure 12b).

**Figure 13:**
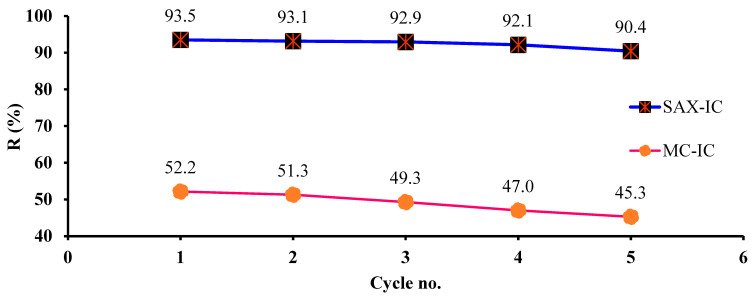
Reusability experiments of IC-MC and IC-SAX for Cu^2+^ adsorption; 0.05 g (0.73 mg IC/g MC and 1.3 mg IC/g SAX), *C_i_* = 7 mg/L, *V* = 0.01 L, 1 h, and 175 rpm (T = 25 ± 2 °C).

The reuse of recycled IC-MC and IC-SAX is a very important step for evaluating their sustainability. Therefore, IC-MC and IC-SAX were employed in five adsorption/desorption cycles, as is presented in Figure 13. In addition, for the last adsorption cycle, adsorption efficiently *R* (%) decreased by 7% for MC-IC and by 3% for SAX-IC compared with the first adsorption/desorption cycle.

To conclude, the complexing polymers exhausted with Cu^2+^ ions were regenerated with HCl, and they were observed to be a sustainable desorption agent for these aims. The regeneration of the complexing polymers exhausted with Cu^2+^ ions was carried out without losing its properties, an important aspect based on the economic point of view of the developed materials. At the same time, they exhibited good reutilization for another adsorption/desorption cycle. Also, the regeneration and reuse of complexing material is an important attribute for recommendations in future environmental applications. Herein, the circular economy approach principal is aimed at developing bio-adsorbents, being more efficient, and reducing waste production.

### 3.8. Applications of Multiple Divalent Metal Ions on IC-MC and IC-SAX Adsorption

The adsorption capacity of IC-MC and IC-SAX for the removal of Ni^2+^, Pb^2+^, and Cu^2+^ from real samples was studied. The simultaneous adsorption of Ni^2+^, Pb^2+^, and Cu^2+^ on IC-MC and IC-SAX are presented in Table 2. The adsorption capacity for divalent metal ions respects the following sequence: Cu^2+^ > Pb^2+^ > Ni^2+^ for tap water spiked for both materials tested.

Regarding tannery wastewater, the following affinity for IC-MC and IC-SAX was observed Cu^2+^ > Ni^2+^ > Pb^2+^. Also, Ni^2+^, Pb^2+^, and Cu^2+^ have the same charge and the tendency to be adsorbed depending on the ionic radius following the order Ni^2+^ (0.70 Å) < Cu^2+^ (0.72 Å) < Pb^2+^ (1.32 Å). This means that the divalent metal ions with the smallest ionic radius can easily diffused into porous structures of complexing polymers and are involved in the complex reaction with complexing functional groups that exist in the structure of modified polymers [30].

Also, the results demonstrate that both IC-MC and IC-SAX are promising materials for simultaneous metal removal, one in the presence of the other from wastewater samples with good adsorption capacity. When the initial concentration of metal ions is increased, an enhanced interaction between metals and complexing polymers was obtained for tap water spiked with both complexing polymers. Also, the percentage removal *R* (%) was more than 85% for all metals studied, results that are dependent on the metal ion concentration existing in tap water spiked.

It was also observed that the addition of complexing polymers reduces the intake of metals by wastewater treatment plants, with a 15.9% Ni^2+^, 15% Pb^2+^, and 78% Cu^2+^ reduction when IC-MC is used. Also, the IC-SAX resin was also employed to remove Ni^2+^, Pb^2+^, and Cu^2+^ from tannery wastewater. It was determined that the adsorption capacity ranged from 0.1 to 0.21 mg/g of metals, indicating an over 55% rate of metal ions adsorption on the tested adsorbent.

To conclude, for equal or variable concentrations of divalent metal ions, complexing polymers show preferential affinity for Cu^2+^ ions in the presence of Pb^2+^ and Ni^2+^.

## 4. Conclusions

In this paper, a new approach for obtaining IC-MC and IC-SAX materials was proposed. In the present study, MC and SAX polymers were chosen, taking into account the following aspects: maize stalk is a natural polysaccharide, inexpensive, and found in abundance after harvesting agricultural crops. At the same time, resin based on styrenedivinyl benzene has good mechanical and chemical stability, being sustainable for several cycles of adsorption/desorption studies. In addition, the chemical structure of those favors IC adsorption. However, experimental conditions for obtaining complexing materials were studied by varying the initial concentration of IC for adsorption in both materials. At the same time, their stability in acid and basic medium was tested.

It was found that the complexing materials are capable of retaining Cu^2+^ ions from aqueous matrices under alkaline pH conditions. The amount of IC retained on MC and SAX significantly improved the adsorption capacity of IC-MC and IC-SAX for Cu^2+^ ions adsorption. It was found that the adsorption capacity of the non-modified MC was 0.3 mg Cu^2+^/g MC, increasing after IC adsorption to 0.65 mg Cu^2+^/g IC-MC when the following experimental conditions were applied *C_i_* = 5 mg/L, 0.05 g IC-MC, 1 h, and 175 rpm (T = 25 ± 2 °C). IC-SAX retains up to four times more than the non-modified resin. So, a significant behavior of adsorption capacity was obtained for both materials after modification. Complexing polymers showed the best Cu^2+^ adsorption at optimum pH = 10 when a maximum adsorption capacity of 0.39 mg/g for IC-MC and 0.78 mg/g for IC-SAX. It was also found that the concentration gradient influences the absorption of Cu ions on both complexing materials.

The results of desorption studies showed that 0.5 M HCl was sufficient for the elution Cu^2+^ ions with a recovery percentage of more than 90% for both complexing polymers. Also, IC-MC and IC-SAX exhibit good selectivity for Cu^2+^ ions in the presence of Ni^2+^ and Pb^2+^ ions in wastewater samples.

Moreover, these complexing materials present numerous benefits, are simple to prepare, eco-friendly, and advantageous if we refer to their recyclability for application in a new adsorption/desorption process.

## Figures and Tables

**Table 1:** Use of chelating natural and synthetic polymers for the removal of Cu^2+^ ions.

Type of Materials	Functionalization Agents	Metal Ions Removed	Optimum Conditions	Adsorption Capacity (mg/g)	References
Kaolin	Amine groups	Cu^2+^	Adsorbent: 10 mg; concentration: 10 mg/L Cu^2+^; contact time: 60 min, temperature: room temperature	61	[39]
Lignocellulose biomass	Ethylenediaminetetraacetic acid (EDTA)	Cu^2+^	Adsorbent: 0.008 g; concentration: 100 mg/L Cu^2+^; contact time: 30 min, temperature: room temperature; pH: 6	157	[40]
Cellulose	p-Aminobenzoic acid	Cu^2+^	Adsorbent: 30 mg; concentration: 200 mg/L Cu^2+^; contact time: 60 min, temperature: room temperature; pH: 6	87	[41]
Straw	Polyethyleneimine hydrochar	Cu^2+^	Adsorbent: 50 mg; concentration: 20 mg/L Cu^2+^; contact time: 50 min, temperature: room temperature; pH: 5.5	56.1	[42]
Maize stalk	Indigo carmine	Cu^2+^	Adsorbent: 50 mg; concentration: 7 mg/L Cu^2+^; contact time: 60 min, temperature: room temperature; pH: 10	0.73	This study
Amberlite XAD-16	Dipicolylamine	Cu^2+^	Adsorbent: 0.1 g; concentration: 300 mg/L Cu^2+^; contact time: 150 min, temperature: room temperature; pH: 5.5	36.6	[43]
Amberlite XAD-4	1,8-Diamino naphthalene	Cu^2+^	Adsorbent: 0.1 g; concentration: 0.02 mg/L Cu^2+^; contact time: 240 min, temperature: room temperature; pH: 7.0	13.8	[44]
Amberlite XAD-4	Calcein blue	Cu^2+^	Adsorbent: 0.05 g; concentration: 100 mg/L Cu^2+^; contact time: 240 min, temperature: room temperature; pH: 6.0	27	[45]
Amberlite IRA 402	Direct Red 23	Cu^2+^	Adsorbent: 0.5 g; concentration: 0.6 mg/L Cu^2+^; contact time: 60 min, temperature: room temperature; pH: 2.5	0.01	[46]
Amberlite XAD7	Direct Red 23	Cu^2+^	Adsorbent: 0.5 g; concentration: 0.6 mg/L Cu^2+^; contact time: 60 min, temperature: room temperature; pH: 2.5	0.02	[46]
Amberlite IRA 402	Indigo carmine	Cu^2+^	Adsorbent: 50 mg; concentration: 50 mg/L Cu^2+^; contact time: 60 min, temperature: room temperature; pH: 10	10.8	This study

As can be observed from Table 1, different values of adsorption capacities for modification of natural polymers were reported in the literature, and it was observed that it is different for each case presented [38,39,40,41,42]. This behavior can be explained as follows: (i) Cu^2+^ adsorption is influenced by the chemical structures of natural polymers employed; (ii) the affinity of Cu^2+^ for the functionalization agent; and (iii) experimental parameters. Also, Amberlite-class resins possess high physio-chemical proprieties [44,47], but to enhance metal adsorption, it is necessary to impregnate, coat, or load chelating or complexing ligands, as presented in Table 1.

**Table 2:** Evaluation of IC-MC and IC-SAX complexing polymers for removal of Ni^2+^, Pb^2+^, and Cu^2+^ from real samples.

Metal Ions		IC-MC		IC-SAX	
		Tap water spiked			
	*C_i_* (mg/L)	*Q_e_* (mg/g)	*R* (%)	*Q_e_* (mg/g)	*R* (%)
Ni^2+^	4.6 ± 1.2	1.22	88.0	1.36	98.5
Pb^2+^	4.9 ± 1.5	1.43	97.1	1.40	95.6
Cu^2+^	5.2 ± 0.55	1.41	90.4	1.56	99.9
	*C_i_* (mg/L)	Tannery wastewater			
Ni^2+^	1.24 ± 0.88	0.03	15.9	0.10	56.0
Pb^2+^	0.79 ± 1.7	0.02	15.0	0.07	55.2
Cu^2+^	1.72 ± 0.95	0.20	77.9	0.21	80.8

## Data Availability

Data are available upon request from the authors.
